# The Effect of the Incorporation Level of *Rosa rugosa* Fruit Pomace and Its Drying Method on the Physicochemical, Microstructural, and Sensory Properties of Wheat Pasta

**DOI:** 10.3390/molecules30153170

**Published:** 2025-07-29

**Authors:** Grażyna Cacak-Pietrzak, Agata Marzec, Aleksandra Rakocka, Andrzej Cendrowski, Sylwia Stępniewska, Renata Nowak, Anna Krajewska, Dariusz Dziki

**Affiliations:** 1 Department of Food Technology and Assessment, Institute of Food Sciences, Warsaw University of Life Sciences, 159C Nowoursynowska Street, 02-776 Warsaw, Poland; grazyna_cacak_pietrzak@sggw.edu.pl (G.C.-P.); andrzej_cendrowski@sggw.edu.pl (A.C.); sylwia_stepniewska@sggw.edu.pl (S.S.); 2 Department of Food Engineering and Process Management, Institute of Food Sciences, Warsaw University of Life Sciences, 159C Nowoursynowska Street, 02-776 Warsaw, Poland; agata_marzec@sggw.edu.pl; 3 Faculty of Food Technology, Warsaw University of Life Sciences, 159C Nowoursynowska Street, 02-776 Warsaw, Poland; ola.rakocka@gmail.com; 4 Department of Pharmaceutical Botany, Medical University of Lublin, 1 Chodźki Street, 20-835 Lublin, Poland; renata.nowak@umlub.pl; 5 Department of Thermal Technology, Lublin University of Life Sciences, Głeboka 31 Street, 20-612 Lublin, Poland; anna.krajewska@up.lublin.pl

**Keywords:** convective drying, microwave–vacuum drying, cooking properties, texture, color, microstructure, antioxidant properties, quality, wheat pasta

## Abstract

This study investigated the effects of the addition of *Rosa rugosa* fruit pomace and drying methods on the properties of pasta, such as culinary properties, color, texture, microstructure, phenolics, antioxidant capacity, and sensory properties. In laboratory conditions, the pasta was produced using low-extraction wheat flour with the addition of pomace at 0, 2, 4, 6, and 8% (g/100 g flour) and dried using either convective or microwave–vacuum drying. The incorporation of pomace into the pasta caused a notable reduction in lightness and increased redness and yellowness, as well as a decrease in pasta hardness and sensory acceptability. The RFP addition also increased the polyphenol content and antioxidant potential. The microwave–vacuum drying resulted in pasta with shorter cooking times, lower cooking loss, and higher total phenolic content and antioxidant activity compared to convective drying. Although the drying method did not markedly affect sensory attributes, ultrastructural analysis revealed that samples subjected to convective drying had a more compact structure, while microwave–vacuum dried pasta exhibited larger pores and smaller starch granules. Total porosity was higher in microwave–vacuum dried pasta. Taking into account both the level of pomace enrichment and the drying technique, the most optimal outcomes were achieved when microwave–vacuum drying was applied and the pomace addition did not exceed 4%.

## 1. Introduction

The genus *Rosa* comprises approximately 120 to 200 species, predominantly native to the temperate regions of the Northern Hemisphere. In Europe, around 30 species are recognized, with 14 to 16 occurring naturally in Poland [1]. Fresh *Rosa rugosa* fruits consist of approximately 60–65% water, 30–40% carbohydrates, and 2–3% protein [2]. The remaining components are present in smaller amounts and include lipids, macro- and microelements (P, K, Ca, Mg, Fe, Cu, Mn), as well as vitamins. Rose fruits are distinguished by their high content of biologically active compounds, including vitamin C, α-tocopherol, polyphenols, and carotenoids [3,4]. Fruits of *Rosa rugosa* find applications in the food sector as ingredients in products like jams, preserves, fruit purees, children's desserts, and syrups. In the pharmaceutical field, they serve as raw materials for vitamin supplements and herbal infusions. Additionally, rosehip seeds have been shown to possess various bioactive effects, including protective benefits for the gastrointestinal system, anti-diabetic activity, anti-aging effects, and immune system support [5,6,7].

Fruit pomace, a by-product of the food industry, represents a highly attractive and cost-effective raw material for use due to its high content of dietary fiber, polyphenols, and other bioactive compounds [8,9]. In particular, pomace from *Rosa rugosa* fruits is gaining popularity as a food-enriching ingredient due to its favorable phytochemical profile and unique antioxidant properties [10,11]. They also exhibit antibacterial activity [12,13]. In recent years, there has been a growing interest in enriching cereal-based products, such as wheat pasta, with natural plant-derived ingredients that can enhance their nutritional value and functional properties [14].

The quality of pasta is determined by the selection of raw materials and the technological process [14,15,16]. The best raw material for pasta production is semolina, due to its high protein content and optimal gluten quality, which contribute to desirable texture and cooking properties. However, common wheat flour is also widely used, primarily because of its greater availability and lower cost. Enriching pasta made from common wheat with plant-based additives can enhance its nutritional value and functional qualities, thereby increasing its appeal to health-conscious consumers [17,18,19]. The most critical unit operation in industrial pasta production is drying, which not only extends the product’s shelf life but also affects its structure, texture, color, nutritional value, and sensory properties [20,21,22]. An alternative to convective drying of pasta is the microwave–vacuum drying method (commonly known as puffing), which involves rapid heating of the material using microwaves under reduced pressure, thereby preventing overheating. Under these conditions, intense water evaporation occurs, significantly shortening the drying time. Importantly, the rapid moisture removal under reduced pressure, and in the absence of oxygen, limits undesirable oxidative changes [23], which is particularly crucial when enriching pasta formulations with heat-sensitive ingredients. Previous studies have shown that MP drying of pasta with added plant protein positively affected the culinary quality, texture, and microstructure of the product. The resulting products exhibited low water activity. High porosity contributed to the rapid hydration of the pasta in boiling water, which allows the product to be considered instant [24]. In industry, MP drying is most often used for drying fruits, vegetables, and cereal grains [17,19,23].

This method is not used for drying pasta. However, the available literature indicates that although the equipment is expensive, the method is energy-efficient due to the short drying time and has a beneficial effect on the final quality of the product without causing it to overheat [25].

The aim of this study was to evaluate the effect of the addition of *Rosa rugosa* fruit pomace (RFP) and different drying methods on the culinary, color, texture, ultrastructure and microstructure, phenolic content, antioxidant capacity, and sensory properties of pasta made from wheat flour.

## 2. Results and Discussion

### 2.1. Basic Chemical Composition of Wheat Flour and Pomace 

Wheat flour (WF), when compared to RFP, exhibited a lower content of protein, fiber, and ash. In contrast, WF contained slightly more fat and over ten times the amount of available carbohydrates (Table 1). The high dietary fiber content in RFP, reaching approximately 62% of dry matter, is particularly noteworthy. Such a substantial proportion of this component indicates the considerable functional potential of this by-product, especially as a food ingredient for dietary fiber enrichment. Dietary fiber plays a key role in regulating digestive processes, stimulates intestinal peristalsis, may contribute to the reduction of blood glucose and cholesterol levels, supports the development of beneficial gut microbiota, and prolongs postprandial satiety [26].

### 2.2. Culinary Properties of Pasta

The experimentally determined cooking time for all convective dried pasta samples was 2.5 min, whereas for pasta dried by the microwave–vacuum method it was shorter—1.5 min (Table S1). The difference in cooking time between convective dried pasta and that dried by the microwave-vacuum method can be attributed primarily to variations in microstructure and the degree of starch gelatinization achieved during drying. In the microwave-vacuum process, rapid vapor expansion under reduced pressure produces a more porous and looser starch-protein matrix, facilitating faster water ingress into the pasta strands. Consequently, a thinner hydration boundary and increased surface area in contact with water enable complete hydration and starch gelatinization in a shorter time [24].

The weight increase index of the analyzed pasta samples ranged from 2.30 (convective drying samples of pasta without RFP to 2.80 for pasta containing 2% RFP, dried using the microwave–vacuum method) (Table 2). The enrichment of pasta with pomace had no significant effect on this parameter. This may be attributed to the fact that the pomace incorporation level used in the formulation (up to 8%) was not sufficient to substantially weaken the compact and well-developed gluten network, which is primarily responsible for limiting excessive water absorption. Slightly higher values of the weight increase index were observed in samples dried by the microwave–vacuum method, although these differences were not statistically significant at the 8% pomace level. The weight increase index reflects the ability of pasta to absorb water during cooking and indicates how many times the mass of the pasta increased after cooking relative to its dry weight. It is a qualitative indicator that indirectly reflects the texture of the final product. Lower values of this index typically suggest that the cooked pasta will be firmer and more compact [27]. The higher values observed for microwave–vacuum dried pasta are likely due to the increased porosity of the product obtained using this method, as well as the puffing effect associated with microwave–vacuum drying [28]. This structure enhances the pasta’s capacity to absorb water during cooking.

Cooking losses (CLs) of pasta were relatively low, ranging from 2.68% to 4.15% (Table 2). Such loss should not exceed 7–8% [29]. The reduction in pasta mass observed during cooking originates chiefly from the solubilization and subsequent diffusion of water-soluble constituents, notably starch polymers, protein fragments, and fine dietary fiber fractions, into the cooking medium. Both the inclusion of *Rosa rugosa* fruit pomace and the drying method significantly influenced dry matter losses during cooking. The addition of pomace increased cooking loss, with higher CL values corresponding to greater pomace levels in the formulation. This effect may be attributed to disruption of the gluten network by the pomace and an increased proportion of water-soluble components, which are more readily leached during cooking. Moreover, pasta dried by the microwave–vacuum method exhibited significantly lower CL than convective drying samples. These findings indicate that this method of drying—performed at lower temperatures and for shorter durations—yields pasta of superior quality and enhanced resistance to nutrients leaching during cooking, most likely owing to better preservation of the colloidal structure of starch and proteins compared to convective drying. Similar observations were reported by Piwińska et al. [15].

### 2.3. Pasta Color

**Figure 1:**
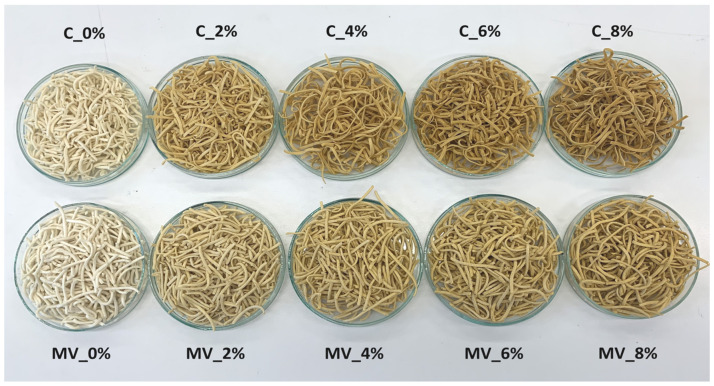
Uncooked pasta with varying contents of *Rosa rugosa* pomace, obtained using convective drying (C) and microwave–vacuum drying (MV).

An analysis of the color parameters of uncooked pasta revealed that both the addition of RFP and the drying method had a significant effect on the lightness (L*), redness (a*), and yellowness (b*) of the product. The addition of pomace had a greater impact on the color of the pasta than the drying method applied. This effect is primarily attributable to the presence of pigments in the pomace, such as anthocyanins, carotenoids, and flavonoids [30]. The L*, a*, and b* values of dried and ground *Rosa rugosa* pomace were 69.6 ± 0.9, 17.4 ± 0.32, and 58.0 ± 1.2, respectively. As a result, its incorporation into the pasta formulation led to a decrease in lightness and an increase in redness and yellowness (Table 3, Figure 1). These differences became more pronounced as the pomace concentration increased. Furthermore, pasta subjected to convective drying was slightly darker and exhibited more intense red and yellow hues compared to that dried using microwave–vacuum technology. These differences may be related to the higher temperature, oxygen exposure, and significantly longer drying time in the convective process, which likely resulted in greater pigment degradation and, consequently, a darker product with enhanced red and yellow tones. Similar findings were reported by other researchers in studies on sour cherry drying [31].

**Figure 2:**
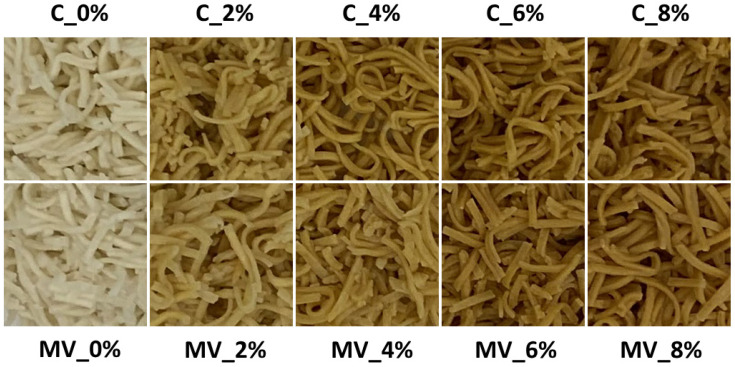
Cooked pasta with varying contents of *Rosa rugosa* pomace, obtained using convective drying (C) and microwave–vacuum drying (MV).

A comparable trend was observed in the cooked pasta samples. After cooking, the samples appeared darker than their uncooked counterparts. In addition, the a* and b* color coordinates increased (Table 4, Figure 2). This is most likely because the color of cooked pasta was measured for the rehydrated product immediately after hydrothermal processing. The darker appearance of cooked pasta compared to the dry product can be attributed to hydration, a decrease in lightness, and pigment changes occurring during thermal treatment. Additionally, the presence of surface moisture influences light reflection, enhancing the perception of a more intense color. The dissolution and migration of natural pigments derived from *Rosa rugosa*, which may concentrate on the surface of the pasta during cooking, also plays an important role. Although convective drying pasta exhibited a similar L* value to that dehydrated using microwave–vacuum drying, it showed significantly higher a* and b* values. These findings are consistent with those reported by other researchers, who emphasize that the drying method plays a key role in determining pasta color [15]. The increased a* and b* values in cooked pasta subjected to convective drying could be explained by its more compact structure, unaffected by puffing. This denser matrix likely restricted pigment movement and loss during cooking, thereby maintaining stronger red and yellow hues. Conversely, the porous texture formed during microwave–vacuum drying may have promoted pigment dispersion and dilution in the cooking medium, resulting in reduced color intensity [24].

### 2.4. Texture of Cooked Pasta

The addition of RFP resulted in significant changes in pasta texture. A decrease in elasticity, hardness, and gumminess was observed, with the extent of these changes increasing proportionally to the pomace content in the formulation. In contrast, the drying method had no effect on texture parameters. At the same level of pomace addition, pasta dried using convective drying exhibited similar values of hardness, elasticity, and chewiness compared to pasta dried by the microwave–vacuum technique (Table 5). The texture of cooked pasta is the result of multiple interacting factors, including the strength and integrity of the gluten network, the degree of starch gelatinization, the water absorption capacity of the dough, the presence of non-gluten components such as dietary fiber or polyphenols, and processing conditions such as drying and cooking parameters [32]. The water content in cooked pasta is also a critical factor [33].

The reduction in pasta hardness following pomace incorporation is primarily attributed to the dilution and weakening of the gluten network, as well as potential interactions between phenolic compounds and proteins. Polyphenols present in rosehip pomace may interact with gluten through several mechanisms, including disulfide bond reduction, alterations in hydrophobic interactions, and protein aggregation [34,35], ultimately leading to the weakening of the pasta structure.

### 2.5. Ultrastructure of Pasta

**Figure 3:**
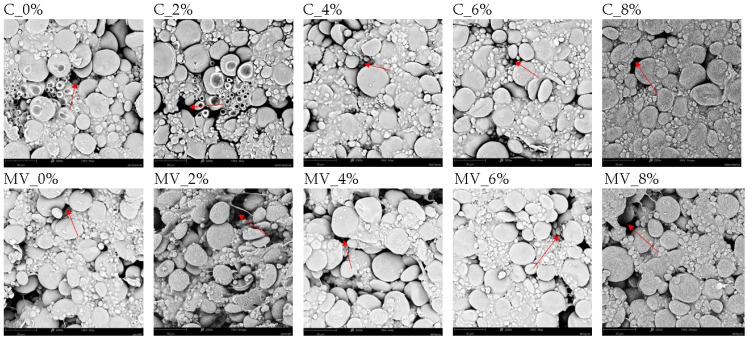
Images from electron microscopy (2000× magnification) of pasta with varying contents of *Rosa rugosa* pomace, obtained using convective drying (C) and microwave–vacuum drying (MV). The red arrow indicates the pores.

In all examined pasta samples, scanning electron microscopy images (Figure 3) revealed aggregates of starch granules with spherical or ellipsoidal shapes embedded within the protein matrix, as well as void spaces (pores). A variation in starch granule size was observed (Table 6). Starch, as the main component of wheat flour and pasta, plays a crucial role in determining the mechanical properties of the product [36]. Notably, starch from soft wheat can reduce the quality of cooked pasta [37]. Furthermore, that parameters describing starch such as diameter, surface area, Feret diameter, and shape factor depended on both the amount of RFP added and the drying method. Microwave–vacuum dried pasta contained significantly smaller starch granules, with reduced Feret diameter and shape factor values, compared to convective dried samples. This effect may be attributed to the lower temperature and reduced pressure conditions during microwave–vacuum drying. Pasta dough dried using convective and microwave–vacuum drying contained the same initial amount of water. During microwave–vacuum drying, pasta is heated rapidly and uniformly using microwaves, and the reduced pressure causes rapid evaporation of water. Starch granules do not have time to hydrate. The microwave–vacuum drying time was 250 seconds. Convective drying at 60 °C involves a long drying time (8 h), during which water initially evaporates from the pasta's surface and subsequently migrates from the center to the surface. During low-temperature convective drying, water transport is slower than during high-temperature drying [38]. The slow water evaporation process could have caused the starch granules to swell, which, after drying, resulted in them being larger than those in the microwave–vacuum dried pasta.

Masato et al. [20] reported that higher drying temperatures resulted in a decreased proportion of small starch granules in pasta. The addition of 2% and 6% RFP led to an increase in these parameters, regardless of the drying method. Smaller starch granules are beneficial for the quality of cooked pasta, as they contribute to reduced cooking loss [39,40]. Due to their larger specific surface area, small starch granules are more effectively retained within the protein (gluten) matrix compared to larger starch granules [20].

### 2.6. X-Ray Microtomography Results of Pasta Samples

**Figure 4:**
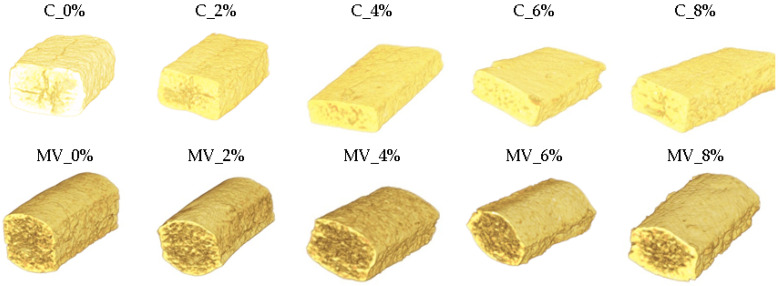
3D projection of pasta with varying contents of *Rosa rugosa* pomace, obtained using convective drying (C) and microwave–vacuum drying (MV).

**Figure 5:**
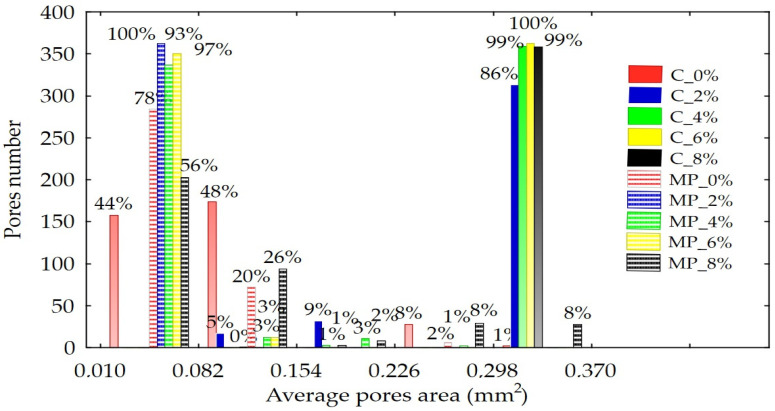
The distribution of pore size in the microstructure of pasta with varying contents of *Rosa rugosa* pomace, obtained using convective drying (C) and microwave–vacuum drying (MV).

The addition of RFP and both drying methods influenced the microstructure of the pasta (Figure 3). The addition of RFP resulted in a more compact microstructure, but only in the convective dried samples. In contrast, pasta dried by microwave–vacuum showed a loosening of the structure due to the addition of pomace (Figure 4). These observations were confirmed by 2D and 3D microstructural analyses using X-ray microtomography (Figure 4 and Figure 5; Table 6). A similar pattern was observed in microwave–vacuum dried pasta enriched with pea protein [24]. It was demonstrated that the addition of alternative pea protein to wheat flour disrupted the protein-starch matrix of the pasta, resulting in a porous microstructure. Gallo et al. [41] showed that the presence of dietary fiber altered the integrity of the protein network in cooked samples. Samples dried by convection exhibited a more compact microstructure compared to those dried by microwave–vacuum, which were characterized by larger void spaces. The rapid water evaporation occurring during microwave–vacuum drying induces a puffing effect, leading to increased porosity of the pasta [24].

Figure 5 and Table S2 present the distribution of pore surface area sizes observed in the samples. Pores were identified based on the analysis of 2D images obtained via X-ray microtomography. Convective dried pasta without pomace addition contained 44%, whereas microwave–vacuum dried pasta contained 78% of pores with a surface area smaller than 0.082 mm^2^. Convective dried pasta with the addition of RFP contained between 86% and 100% large pores, with surface areas exceeding 0.298 mm^2^. In contrast, microwave–vacuum dried pasta with RFP addition contained pores with surface areas smaller than 0.082 mm^2^. The most heterogeneous pore surface area distribution was observed in pasta with an 8% RFP addition dried by microwave–vacuum, which contained both small and large pores.

The level of added RFP and the drying method significantly influenced the 3D structural parameters of the pasta (Table 7). The percent of object volume (POV), representing the proportion of solid material in the analyzed volume, was significantly higher in convection-dried pasta than in microwave–vacuum dried samples. In contrast, an opposite trend was observed for total porosity. Among the convection-dried samples, the control (without pomace addition) exhibited the lowest POV and the highest total porosity, reaching 30.86%. The incorporation of RFP at levels ranging from 2% to 8% resulted in a significantly higher POV and lower total porosity, ranging from 2.30% to 1.60%. In microwave–vacuum dried pasta, POV values for the control, 2%, and 4% RFP samples were similar, with total porosity values around 50%. A significant increase in POV and a decrease in total porosity (to approximately 35%) were only observed at 6% and 8% RFP addition. All pasta samples exhibited a low proportion of closed pores and were characterized by predominantly open porosity. The structure thickness (St.Th) of the control pasta did not differ significantly between the drying methods. However, the combination of drying method and RFP addition led to significant differences in this parameter. Conventionally dried pasta enriched with RFP showed significantly higher St.Th values than microwave–vacuum-dried samples containing the same additive. The degree of anisotropy was primarily influenced by the drying method. Convection drying resulted in a higher degree of anisotropy compared to microwave–vacuum drying.

### 2.7. Phenolics Content and Antioxidant Capacity

Table 8 presents the total phenolic content and antioxidant activity of extracts obtained from the analyzed pasta samples. Both the drying method and the addition of RFP significantly affected the phenolic content and antioxidant potential of the samples. Pasta dried using the microwave–vacuum method exhibited a higher phenolic content and lower EC50 values (indicating stronger antioxidant activity) compared to convective dried pasta. The higher phenolic content and stronger antioxidant activity (indicated by lower EC50 values) observed in pasta subjected to microwave–vacuum drying may be attributed to the shorter drying time, reduced oxygen exposure, and lower processing temperature, all of which help minimize the degradation of thermolabile and easily oxidizable bioactive compounds. Additionally, microwave–vacuum drying better preserves the cellular structure, which may contribute to enhanced retention of phenolic compounds. These findings are consistent with literature reports indicating that microwave–vacuum drying is more effective in preserving antioxidants compared to convective drying methods [31].

Enrichment of pasta with fruit pomace resulted in increased antioxidant activity and total phenolic content, with higher values corresponding to greater levels of pomace addition. In pasta containing 8% pomace, the phenolic content more than doubled compared to the control sample without pomace addition. This effect is attributable to the high concentration of phenolic compounds in the fruit pomace itself, which, in the case of the applied *Rosa rugosa* pomace, amounted to 26.82 ± 1.08 mg GAE (gallic acid equivalents) per gram of d.m. (dry matter).

### 2.8. Sensory Properties

Table 9 presents the results of the sensory evaluation of the pasta samples. In most cases, the drying method did not exert a statistically significant effect on the assessed attributes. In contrast, the addition of *Rosa rugosa* fruit pomace (RFP) had a marked influence on the appearance, taste, aroma, and texture of the pasta. The appearance of pasta enriched with RFP was rated more favorably due to its color, which was considered more acceptable compared to that of the control sample. Pasta made from common wheat flour typically has a lighter, grayish-cream hue, which is often perceived as less visually appealing. In contrast, pasta made from semolina is characterized by an intense yellow color, commonly associated with high quality and freshness, and is generally more attractive to consumers. Even a 2% addition of RFP significantly altered the product’s color, notably increasing the yellowness of the samples, which positively influenced consumer acceptance of this attribute. The dominant pigments in RFP are anthocyanins, primarily cyanidin-3-O-glucoside and pelargonidin-3-O-glucoside, which are responsible for the intense red to purple coloration of the fruit. In addition to anthocyanins, carotenoids such as β-carotene and lycopene are also present, contributing yellow to orange hues, although typically in lower concentrations than anthocyanins [42]. However, the acceptability of other attributes such as taste, aroma, and texture was substantially reduced, particularly when the RFP addition reached 6% and 8% of the flour weight. RFP is characterized by a strong, slightly acidic, and often herbal-floral aroma, which may overpower the neutral flavor profile of conventional pasta. It may also impart a bitter or astringent aftertaste, especially at higher inclusion levels, due to the presence of significant amounts of polyphenolic compounds, including tannins [13]. Regarding texture, a decline in firmness and an increase in stickiness of the cooked pasta were observed. This was likely due to the disruption and weakening of the gluten network, a finding also confirmed by instrumental texture analysis described in Section 2.1, where increasing levels of RFP resulted in decreased elasticity and hardness of the pasta. Similar results were obtained by other authors who incorporated various plant-based additives into pasta formulations [14,43]. In summary, the highest overall acceptability was observed for the control sample and for pasta containing 2% and 4% RFP. By contrast, formulations with 6% and 8% RFP were not accepted by consumers.

### 2.9. Correlation Analysis

**Figure 6:**
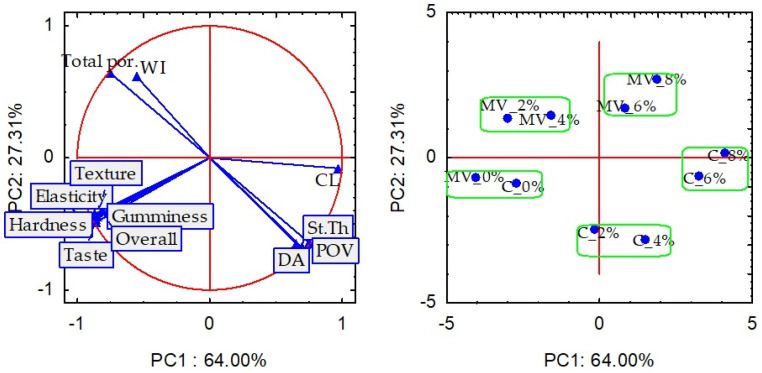
PCA diagram. Culinary parameters—Left panel: PCA biplot showing the relationships between quality attributes of pasta, right panel: Distribution of pasta samples with different levels of RFP addition in the PCA space: WI—weight increase index; Cl—cooking loss; Textural parameters: elasticity, hardness, gumminess; Structural parameters: POV, percent object volume; Total por.—total porosity; St.Th—structural thickness; DA—degree of anisotropy; Sensory parameters: texture; taste, overall quality.

To illustrate the relationships among the analyzed culinary, textural, structural, and sensory parameters and the pasta samples containing Rosa rugosa fruit pomace (RFP), dried using convection and microwave–vacuum methods, principal component analysis (PCA) was conducted. The first two principal components (PCs) explained 91% of the total variance in the measured parameters. Specifically, PC1 and PC2 accounted for 64.0% and 27.3% of the total variance, respectively. PC1 was primarily associated with cooking loss (CL), textural parameters (elasticity, hardness, gumminess), and sensory attributes (taste, texture, overall acceptability), while PC2 was related to whiteness index (WI) and structural parameters, including percent object volume, total porosity, structure thickness (St. Th), and degree of anisotropy (DA). The pasta samples without RFP, dried using convection and microwave–vacuum methods, were clustered together on the left side of the PCA plot (Figure 6). This indicates that the samples shared similar characteristics, such as the lowest cooking loss, favorable texture, and the highest sensory scores. These samples were porous but exhibited a low degree of anisotropy. Pasta samples with 2% and 4% RFP, dried using microwave–vacuum drying, exhibited the most favorable texture and sensory properties, and their porosity was comparable to that of the samples without RFP. However, all RFP-containing samples that were convection-dried, as well as those with 6% and 8% RFP dried using microwave–vacuum drying, were positioned on the right side of the PCA plot, indicating inferior texture and lower sensory scores.

Significant correlations were found between the instrumentally measured texture and sensory properties (Table S3). Strong correlations were also observed between culinary parameters and microstructure, demonstrating that culinary parameters are strongly influenced by the product's microstructure. No correlation was observed between the texture and microstructure of the paste. This is likely because the texture of cooked pasta was measured, while the microstructure of dry pasta was measured. This implies a need for further research to compare the texture and microstructure of cooked pasta. 

## 3. Materials and Methods

### 3.1. Material

Wheat flour type 450, purchased from a local store in Warsaw, was produced industrially by Polskie Młyny Sp. z o.o. (Szymanów, Poland). The pomace was a by-product obtained after juice pressing of *Rosa rugosa* fruit. The fruits were sourced from a plantation located in the Kłodzka Valley (Kłodzko, Poland). After juice pressing, the pomace was frozen and stored at −18 °C. The freeze-drying process was conducted using a tabletop freeze dryer Alpha 1–4 LSCplus (Martin Christ Gefriertrocknungsanlagen GmbH, Osterode am Harz, Germany) for 45 hours at −56 °C and a pressure of 1.03 mbar. The obtained dry material was ground in a Grindomix GM 200 homogenizer (Retsch GmbH, Haan, Germany), then ground material (particle < 0.3 mm) was transferred into bags, vacuum-sealed, and stored in a dark place until analysis. The basic chemical composition of flour and pomace was analyzed [43]. All determinations were carried out in three replicates. 

The following chemicals were used in the study: Folin–Ciocalteu reagent, ABTS (2,2′-azino-bis(3-ethylbenzothiazoline-6-sulfonic acid)), DPPH (2,2-diphenyl- 1-picrylhydrazyl), methanol, and sodium carbonate, all purchased from Sigma-Aldrich (Poznań, Poland).

### 3.2. Pasta Preparation

Pasta samples were prepared from WF and water, and from mixtures of wheat flour with freeze-dried RFP and water. Wheat flour was partially replaced with pomace at levels of 2, 4, 6, and 8%. A fixed amount of water (37%) was added to both the pure flour and the flour–pomace mixtures. The dough was mixed using a T-5KPM5EER mixer (KitchenAid, Benton Harbor, MI, USA) for 5 min. Subsequently, the dough was rolled into sheets with a thickness of 1.5 mm and cut into tagliatelle using a 5KSMPSA pasta roller and cutter (KitchenAid, Benton Harbor, USA). The formed pasta was dried by two methods: convective drying and microwave–vacuum drying.

For convective drying, weighed portions of pasta (100 g) were placed on perforated trays and dried at 60 °C for approximately 8 h in a SUP-65 convective dryer (WAMED, Warsaw, Poland). It was assumed that the pasta moisture content after drying should be 12.0% (±0.2%) [44].

In the microwave–vacuum drying method, weighed portions of pasta (100 g) were placed in a chamber of a laboratory microwave–vacuum dryer (Promis-Tech, Wroclaw, Poland). Drying was conducted at 40 °C for 250 s under a pressure of 55 hPa with a microwave power of 450 W. This was followed by a stabilization period lasting 200 s. Drying parameters were determined experimentally, assuming a target moisture content of 12.0% (±0.2%) after drying [14].

After cooling, the dried pasta samples were packed in paper bags and placed in a tightly closed plastic box. The samples were stored in a dark place at temperature 22 ± 2 °C until analysis.

### 3.3. Measurement of Culinary Properties of Pasta

The cooking time, weight increase index, and dry matter loss during cooking were determined as described by Sujka et al. [44]. All determinations were carried out in three replicates.

### 3.4. Color Coordinates

The color coordinates of both raw and cooked pasta were measured using a CR-200 spectrophotometer (Konica Minolta, Tokyo, Japan) in the CIE Lab* color space, according to the established methodology. The following color parameters were determined: lightness (L*), a* (positive values indicate redness, negative values indicate greenness), and b* (positive values indicate yellowness, negative values indicate blueness) [45]. The total color difference (ΔE) between the control sample without RFP addition and pasta samples enriched with RFP was also calculated [41]. All measurements were performed in ten replicates. 

### 3.5. Texture Measurement

The texture of the pasta samples was determined using a TA-XT2i texture analyzer (Stable Micro Systems, London, UK) employing the Texture Profile Analysis (TPA) method according to Espinosa-Solis et al. [46]. Cooked pasta was placed in plastic containers with a diameter of 55 mm and a height of approximately 25 mm, and subjected to two consecutive compressions using a cylindrical probe with a diameter of 25 mm at a test speed of 1 mm/s. The interval between the two compressions was 10 seconds. Texture parameters, including cohesiveness, hardness, and chewiness were calculated using the Texture Expert Exceed software. Eight replicates were analyzed per sample. 

### 3.6. Ultrastructural Analysis by Scanning Electron Microscopy

Dry pasta samples were sputter-coated with gold (200 Å) in a Leica sputter coater (Leica Mikrosysteme, Veinna, Austria), yielding a coating thickness of 5 nm. The coated specimens were examined using a Phenom ProX scanning electron microscope (Phenom-World B.V., FEI Company, Eindhoven, the Netherlands) at an accelerating voltage of 10 kV and a magnification of 2000×.

The resulting micrographs of pasta cross-sections were processed in the MultiScan image-analysis software (v13.11 CSS Scan). Starch-granule boundaries were delineated manually to generate regions corresponding to individual granule cross-sectional areas. From these two-dimensional sections, the following cellular parameters and their distributions were determined: area (S, μm^2^), perimeter (L, μm), and Feret diameters (H and V, μm). The Feret aspect ratio (WF = H/V) and shape factor (WK = (4πS/L^2^)) were then calculated. For each pasta sample, 60 starch granules were analyzed.

### 3.7. Microstructure Analysis by X-Ray Microtomography

Pasta specimens were scanned using a SkyScan 1272 micro-CT system (Bruker-microCT, Kontich, Belgium). A 180° rotational scan was performed with a 0.4° rotation step and four-frame averaging at each angular position. Each pasta variant was scanned in duplicate; sample lengths were approximately 20 mm. The source–object–detector distance was adjusted to yield an isotropic voxel size of 20 µm. Scans were acquired at 50 kV and 200 µA without filtration, using a CCD camera with a 9 µm pixel resolution. A total of 470 cross-sectional images were recorded per scan.

Image reconstruction was carried out in NRecon v1.6.9.8 (Bruker-microCT, Kontich, Belgium) with Gaussian smoothing and ring-artifact correction. The resulting dataset comprised 180 grayscale tomographic slices (values 30–180), forming a three-dimensional volume. For quantitative analysis, images were cropped to a 25 × 37 pixel region of interest (ROI) in CTAn v1.9.3.3 (Bruker-microCT, Kontich, Belgium). The 2D stack was used to reconstruct the 3D volume, and measurements were performed on volumes of interest (VOI) of 1.35 mm^3^.

The following three-dimensional parameters were calculated:

- percent object volume (%): total volume of binarized pasta structures within the VOI.
- total porosity: proportion of void space within the VOI.
- structure thickness: average thickness of pasta strands.
- degree of anisotropy: measure of directional dependency of structural features.

For realistic visualization of the reconstructed microstructure, CTvol software (Bruker-microCT, Kontich, Belgium) was employed.

### 3.8. Antioxidant Properties of Extracts from Pasta Samples

To determine the total polyphenol content and antioxidant activity, methanolic extracts were prepared from pasta samples according to the methodology described by Sujka et al. [44].

The total phenolic content was determined using the Folin–Ciocalteu method and expressed as GAE (gallic acid equivalents) per gram of dry matter (d.m.) [44]. Antioxidant activities (DPPH and ABTS) were measured using an Epoch2TC microplate spectrophotometer (BioTek Instruments Inc., Winooski, USA). The ABTS radical scavenging activity was assessed following the methodology of Re et al. [47], with slight modifications [46]. The DPPH radical scavenging activity was determined according to the method described by Brand-Williams et al. [48]. The radical scavenging activities of the extracts were expressed as the concentration required to reduce the initial ABTS and DPPH radical concentrations by 50% (EC50 value).

### 3.9. Sensory Evaluation

A sensory analysis was conducted using a nine-point hedonic scale, where 1 denoted “extremely undesirable,” 5 denoted “neither desirable nor undesirable,” and 9 denoted “extremely desirable” [49]. The panel comprised 46 untrained assessors (29 women and 17 men; staff and students of Warsaw University of Life Sciences) aged 20–55 years. Participants were selected based on self-declared good health status, absence of gluten allergy, and regular consumption of pasta. The attributes evaluated were taste and aroma, color, texture, and overall acceptability. Assessments were performed under white lighting in a room maintained at 22 °C.

### 3.10. Statistical Analysis

Statistical analysis of the obtained data was performed using Statistica 13.3 software (TIBCO Software, Palo Alto, CA, USA). A two-way analysis of variance (ANOVA) was conducted, and homogeneous groups were determined by Tukey’s test. In addition, the *t*-test was used to compare the chemical composition of wheat flour and *Rosa rugosa* fruit pomace. The significance level of α = 0.05 was applied. Principal component analysis (PCA) was performed, as well as Pearson correlation coefficients.

## 4. Conclusions

The conducted study demonstrated that both the drying method and the enrichment of pasta with *Rosa rugosa* pomace had a significant impact on several pasta properties. Compared to microwave–vacuum-dried pasta, convection-dried pasta was characterized by a longer cooking time, a lower weight increase index, and greater cooking loss. The total phenolic content and antioxidant activity were higher in microwave–vacuum-dried pasta samples. However, the drying method did not significantly affect the sensory evaluation results. Ultrastructural analysis revealed that samples dried by convection exhibited a more compact microstructure compared to those dried by microwave–vacuum, which showed larger void spaces. Microwave–vacuum-dried pasta also contained significantly smaller starch granules, as evidenced by lower Ferret diameter and shape factor values compared to the convection-dried samples. 

The addition of pomace led to a decrease in lightness and an increase in the red and yellow color coordinates. It also resulted in increased cooking loss, decreased pasta hardness, and lower sensory scores. On the other hand, total phenolic content and antioxidant activity increased with pomace addition. The most heterogeneous pore surface area distribution was observed in pasta with 8% RFP addition dried by microwave–vacuum, which contained both small and large pores.

Taking into account both the drying method and the level of pomace addition, the most favorable results were achieved using microwave–vacuum drying combined with pomace content not exceeding 4%.

## Figures and Tables

**Table 1:** Basic chemical composition of WF and RFP (% dry mass).

Raw Material	Protein	Fat	Insoluble Fiber	Soluble Fiber	Ash	Carbohydrates
WF	9.62 ± 0.12^a^	0.67 ± 0.04^b^	1.43 ± 0.07^a^	0.20 ± 0.01^a^	0.47 ± 0.03^a^	87.61
RFP	14.30 ± 0.25^b^	0.37 ± 0.05^a^	45.26 ± 0.52^b^	16.05 ± 0.29^b^	15.54 ± 0.08^b^	8.48

^a,b^—homogeneous groups (*p* < 0.05); WF—wheat flour, RFP—*Rosa rugosa* fruit pomace.

**Table 2:** Weight increase index and cooking loss of pasta with varying contents of *Rosa rugosa* pomace, obtained using convective drying (C) and microwave–vacuum drying (MV).

Pomace Content (%)	Drying Method	Weight Increase Index (-)	Cooking Loss (%)
0		2.45 ± 0.07^abc^	3.13 ± 0.11^c^
2		2.50 ± 0.10^bc^	3.37 ± 0.06^d^
4	C	2.30 ± 0.00^a^	3.64 ± 0.08^ef^
6		2.37 ± 0.06^ab^	3.85 ± 0.13^f^
8		2.50 ± 0.10^bc^	4.15 ± 0.09^g^
0		2.57 ± 0.06^cd^	2.68 ± 0.11^a^
2		2.80 ± 0.10^e^	2.82 ± 0.04^b^
4	MV	2.70 ± 0.20^de^	3.10 ± 0.05^bc^
6		2.53 ± 0.06^c^	3.55 ± 0.07^de^
8		2.53 ± 0.06^c^	3.50 ± 0.02^de^
**Two-factor analysis of variance**			
**Factor**		***p*** **-value**	
Pomace content		0.022 *	<0.001 *
Drying method		<0.001 *	<0.001 *
Pomace content × drying method		0.027 *	0.009 *

^a–g^—homogeneous groups (*p* < 0.05); *—statistically significant (*p* < 0.05).

**Table 3:** Color coordinates of uncooked pasta with varying contents of *Rosa rugosa* pomace, obtained using convective drying (C) and microwave–vacuum drying (MV).

Pomace Content (%)	Drying Method	L*	a*	b*	ΔE
0	C	91.21 ± 0.33^g^	−0.50 ± 0.39^a^	12.22 ± 0.74^b^	-
2		84.93 ± 0.31^ab^	2.63 ± 0.16^bc^	25.94 ± 0.27^d^	15.69 ± 0.35^b^
4		84.59 ± 0.20^e^	2.91 ± 0.11^c^	28.94 ± 0.41^e^	18.58 ± 0.45^c^
6		81.44 ± 0.15^b^	4.32 ± 0.10^f^	32.38 ± 0.19^h^	23.19 ± 0.17^e^
8		79.73 ± 0.33^a^	4.84 ± 0.23^g^	33.73 ± 0.59^i^	25.24 ± 0.66^g^
0	MV	92.59 ± 0.10^h^	−0.54 ± 0.06^a^	9.44 ± 0.23^a^	-
2		87.82 ± 0.15^f^	1.31 ± 0.06^b^	22.05 ± 0.38^c^	13.86 ± 0.40^a^
4		84.97 ± 0.34^e^	2.84 ± 0.18^bc^	29.13 ± 0.48^f^	21.65 ± 0.57^d^
6		83.70 ± 0.17^d^	3.23 ± 0.08^d^	31.10 ± 0.33^g^	23.96 ± 0.36^f^
8		82.63 ± 0.19^c^	3.52 ± 0.09^e^	32.14 ± 0.39^h^	25.38 ± 0.42^g^
***Two-factor analysis of variance***					
**Factor**		***p-value***			
Pomace content		<0.001 *	<0.001 *	<0.001 *	<0.001 *
Drying method		<0.001 *	<0.001 *	<0.001 *	<0.001 *
Pomace content × drying method		<0.001 *	<0.001 *	<0.001 *	<0.001 *

^a–i^—homogeneous groups (*p* < 0.05); *—statistically significant (*p* < 0.05).

**Table 4:** Color coordinates of cooked pasta with varying contents of *Rosa rugosa* pomace, obtained using convective drying (C) and microwave–vacuum drying (MV).

Pomace Content (%)	Drying Method	L*	a*	b*	ΔE
0	C	72.63 ± 0.35^g^	−2.04 ± 0.13^a^	13.12 ± 0.63^b^	-
2		63.40 ± 0.99^e^	5.03 ± 0.62^c^	33.09 ± 1.49^d^	23.14^b^
4		58.68 ± 0.20^c^	10.30 ± 1.05^e^	38.99 ± 0.42^f^	31.81^cd^
6		55.26 ± 0.71^b^	12.11 ± 0.54^f^	38.40 ± 0.87^f^	33.79^de^
8		53.29 ± 0.72^a^	12.82 ± 0.41^f^	37.80 ± 0.99^f^	34.70^e^
0	MV	70.81 ± 0.61^f^	−2.23 ± 0.11^a^	11.48 ± 0.53^a^	-
2		62.07 ± 0.79^d^	3.66 ± 0.44^b^	28.42 ± 1.04^c^	19.91^a^
4		57.84 ± 0.90^c^	7.87 ± 0.69^d^	36.10 ± 0.95^e^	29.63^c^
6		55.18 ± 0.65^b^	9.68 ± 0.57^e^	35.64 ± 1.16^e^	30.03^c^
8		53.61 ± 0.48^a^	10.54 ± 0.81^e^	34.83 ± 1.18^e^	31.70^cd^
***Two-factor analysis of variance***					
**Factor**		***p-value***			
Pomace content		<0.001 *	<0.001 *	<0.001 *	<0.001 *
Drying method		<0.001 *	<0.001 *	<0.001 *	*<0.00* 1 *
Pomace content × drying method		<0.001 *	<0.001 *	*0.196*	*0.516*

^a–g^—homogeneous groups (*p* < 0.05); *—statistically significant (*p* < 0.05).

**Table 5:** Texture parameters of cooked pasta with varying contents of *Rosa rugosa* pomace, obtained using convective drying (C) and microwave–vacuum drying (MV).

Pomace Content (%)	Drying Method	Elasticity (-)	Hardness (N)	Gumminess (N)
0		0.69± 0.02^b^	5.62 ± 0.47^c^	3.02 ± 0.23^b^
2		0.63 ± 0.01^ab^	5.26 ± 0.23^bc^	2.78 ± 0.13^b^
4	C	0.66 ± 0.02^ab^	4.84 ± 0.26^bc^	2.81 ± 0.96^b^
6		0.58 ± 0.09^a^	3.93 ± 0.15^ab^	1.84 ± 0.13^a^
8		0.53 ± 0.03^a^	3.03 ± 0.53^a^	1.58 ± 0.21^a^
0		0.73 ± 0.02^b^	5.75 ± 0.41^c^	3.37 ± 0.06^b^
2		0.67 ± 0.03^ab^	4.88 ± 0.52^bc^	2.79 ± 0.15^b^
4	MV	0.64 ± 0.04^ab^	4.93 ± 0.76^bc^	2.58 ± 0.50^b^
6		0.55 ± 0.06^a^	4.43 ± 0.08^ab^	2.09 ± 0.11^a^
8		0.54 ± 0.02^a^	3.47 ± 0.32^a^	1.81 ± 0.08^a^
***Two-factor analysis of variance***				
**Factor**		***p-value***		
Pomace content		0.009 *	0.001 *	<0.001 *
Drying method		*0.807*	*0.705*	*0.485*
Pomace content × drying method		*0.569*	*0.814*	*0.690*

^a–c^—homogeneous groups (*p* < 0.05); *—statistically significant (*p* < 0.05).

**Table 6:** Ultrastructural parameters of starch granules in pasta with varying contents of *Rosa rugosa* pomace, obtained using convective drying (C) and microwave–vacuum drying (MV).

Pomace Content (%)	Drying Method	Surface Area (µm^2^)	Feret Diameter (-)	Shape Factor (-)
0		241.51 ± 182.97^b^	0.860 ± 0.09^b^	0.757 ± 0.072^c^
2		255.28 ± 198.20^b^	0.835 ± 0.128^b^	0.731 ± 0.083^bc^
4	C	218.85 ± 166.09^a^	0.802 ± 0.130^ab^	0.728 ± 0.075^bc^
6		248.85 ± 160.85^b^	0.852 ± 0.123^ab^	0.734 ± 0.085^bc^
8		206.12 ± 172.34^a^	0.809 ± 0.124^ab^	0.735 ± 0.067^bc^
0		239.86 ± 236.83^b^	0.817 ± 0.147^ab^	0.725 ± 0.098^bc^
2	MV	217.27 ± 174.43^ab^	0.803 ± 0.131^ab^	0.711 ±0.096^ab^
4		165.15 ± 118.44^a^	0.765 ± 0.149^a^	0.683 ± 0.102^a^
6		212.37 ± 150.71^ab^	0.795 ± 0.131^ab^	0.703 ± 0.083^ab^
8		168.10 ± 133.72^a^	0.814 ± 0.126^ab^	0.716 ± 0.094^ab^
***Two-factor analysis of variance***				
**Factor**		***p-value***		
Pomace content		0.032 *	0.018 *	0.034 *
Drying method		0.017 *	0.002 *	<0.001 *
Pomace content × drying method		*0.829*	*0.425*	*0.788*

^a–c^—homogeneous groups (*p* < 0.05); *—statistically significant (*p* < 0.05).

**Table 7:** 3D Microstructural parameters of pasta with varying contents of *Rosa rugosa* pomace, obtained using convective drying (C) and microwave–vacuum drying (MV).

Pomace Content (%)	Drying Method		Percent Object Volume (%)	Closed Porosity (%)	Total Porosity (%)	Structure Thickness (mm)	Degree of Anisotropy (-)
0	C		69.14 ± 2.64^b^	0.57 ± 0.15^c^	30.86 ± 2.64^b^	0.090 ± 0.001^a^	1.96 ± 0.15^a^
2			97.70 ± 2.81^c^	0.25 ± 0.07^ab^	2.30 ± 2.81^a^	0.330 ± 0.077^b^	6.11± 2.24^bc^
4			99.49 ± 0.41^c^	0.09 ± 0.00^ab^	0.51 ± 0.41^a^	0.444 ± 0.007^c^	6.85 ± 1.28^c^
6			99.56 ± 0.07^c^	0.19 ± 0.03^ab^	0.44 ± 0.07^a^	0.387 ± 0.022^bc^	5.34 ± 2.40^abc^
8			98.40 ± 1.14^c^	0.29 ± 0.13^ab^	1.60 ± 1.14^a^	0.368 ± 0.067^bc^	6.13 ± 3.20^bc^
0	MV		50.72 ± 6.19^a^	0.20 ± 0.05^ab^	49.28 ± 6.19^c^	0.104±0.006^a^	3.10±0.24^abc^
2			45.97 ± 1.77^a^	0.04 ± 0.04^a^	54.03 ± 1.77^c^	0.082±0.005^a^	2.53±0.31^ab^
4			50.17 ± 0.89^a^	0.18 ± 0.09^ab^	49.83 ± 0.89^c^	0.098±0.007^a^	3.30±0.03^abc^
6			64.78±0.95^b^	0.21±0.00^ab^	35.22±0.95^b^	0.114±0.015^a^	3.17±0.57^abc^
8			64.70±14.56^b^	0.20±0.14^ab^	35.30±14.56^b^	0.105±0.026^a^	2.85±0.05^ab^
***Two-factor analysis of variance***							
**Factor**		***p-value***					
Drying method		<0.001 *		0.016 *	<0.001 *	<0.001 *	0.007 *
Pomace content		<0.001 *		0.012 *	0.001 *	<0.001 *	*0.257*
Pomace content × drying method		0.001 *		0.026 *	0.007 *	<0.001 *	*0.220*

^a–c^—homogeneous groups (*p* < 0.05); *—statistically significant (*p* < 0.05).

**Table 8:** Phenolic content and antioxidant activity of cooked pasta with varying contents of *Rosa rugosa* pomace, obtained using convective drying (C) and microwave–vacuum drying (MV).

Pomace Content (%)	Drying Method	TPC (mg GAE/g d.m.)	EC50_DPPH_ (mg d.m./mL)	EC50_ABTS_ (mg d.m./mL)
0		1.03 ± 0.07^a^	391.1 ± 4.8^g^	645.9 ± 14.6^h^
2		1.45 ± 0.03^b^	369.0 ± 3.5^f^	498.4 ± 8.7^f^
4	C	1.60 ± 0.24^b^	350.6 ± 3.8^e^	458.8 ± 14.9^e^
6		2.07 ± 0.06^c^	253.8 ± 5.9^c^	366.1 ± 10.9^d^
8		2.43 ± 0.05^d^	187.9 ± 0.5^b^	261.0 ± 9.4^b^
0		1.14 ± 0.03^a^	340.8 ± 4.3^e^	537.9 ± 11.0^g^
2		1.66 ± 0.06^b^	322.8 ± 2.4^d^	389.1 ± 5.3^d^
4	MV	2.04 ± 0.01^c^	253.6 ± 8.7^c^	314.1 ± 0.5^c^
6		2.35 ± 0.10^d^	191.5 ± 4.8^b^	273.7 ± 9.2^b^
8		2.77 ± 0.06^e^	156.8 ± 5.8^a^	217.1 ± 5.4^a^
***Two-factor analysis of variance***				
**Factor**		***p-value***		
Pomace content		<0.001 *	<0.001 *	<0.001 *
Drying method		<0.001 *	<0.001 *	<0.001 *
Pomace content × drying method		*0.054*	<0.001 *	<0.001 *

^a–h^—homogeneous groups (*p* < 0.05), TPC–total phenolics content, EC50_DPPH_—effective concentration of the extract required to reduce 50% of DPPH and ABTS free radicals, respectively; *—statistically significant (*p* < 0.05).

**Table 9:** Sensory properties of cooked pasta with varying contents of *Rosa rugosa* pomace, obtained using convective drying (C) and microwave–vacuum drying (MV).

Pomace Content (%)	Drying Method	Appearance (pkt)	Smell (pkt)	Taste (pkt)	Texture (pkt)	Overall Acceptability (pkt)
0	C	6.6 ± 0.8^a^	7.9 ± 0.8^bc^	7.9 ± 0.7^cd^	6.9 ± 0.7^d^	7.2 ± 0.8^e^
2		7.6 ± 0.9^b^	8.3 ± 0.8^c^	7.8 ± 1.0^bcd^	6.7 ± 0.6^cd^	6.9 ± 0.8^e^
4		7.9 ± 0.6^bcd^	8.3 ± 0.8^c^	7.4 ± 1.0^bc^	6.2 ± 0.8^bc^	6.0 ± 0.9^c^
6		8.1 ± 0.7^cd^	7.5 ± 0.9^b^	6.4 ± 0.8^a^	6.0 ± 0.9^ab^	5.3 ± 1.1^ab^
8		8.0 ± 0.7^bcd^	6.5 ± 0.9^a^	6.1 ± 1.0^a^	5.8 ± 1.1^ab^	5.9 ± 1.1^bc^
0	MV	6.4 ± 0.7^a^	7.9 ± 0.8^bc^	8.1 ± 0.6^d^	7.1 ± 0.7^d^	7.1 ± 0.8^e^
2		7.7 ± 0.8^bc^	8.3 ± 0.8^c^	7.6 ± 0.9^bcd^	6.6 ± 0.7^cd^	6.8 ± 0.8^de^
4		8.0 ± 0.5^bdc^	8.3 ± 0.7^c^	7.3 ± 1.0^b^	6.3 ± 0.8^bc^	6.2 ± 0.8^cd^
6		8.2 ± 0.6^d^	7.5 ± 0.9^b^	6.5 ± 0.9^a^	5.9 ± 1.0^ab^	5.9 ± 1.3^bc^
8		7.9 ± 0.9^bcd^	6.6 ± 1.0^a^	6.2 ± 0.9^a^	5.6 ± 1.1^a^	5.2 ± 1.4^a^
***Two-factor analysis of variance***						
**Factor**		***p-value***				
Pomace content		<0.001 *	<0.001 *	<0.001 *	<0.001 *	<0.001 *
Drying method		*0.900*	*0.868*	*0.877*	*0.784*	*0.643*
Pomace content × drying method		*0.665*	*0.969*	*0.729*	*0.690*	<0.001 *

^a–e^—homogeneous groups (*p* < 0.05); *—statistically significant (*p* < 0.05).

## Data Availability

The original contributions presented in the study are included in the article; further inquiries can be directed to the corresponding authors.

## Supplementary Materials

The following supporting information can be downloaded at: https://www.mdpi.com/article/10.3390/molecules30153170/s1, Table S1:Cooking time of pasta with varying levels of *Rosa rugosa* pomace, obtained using convective (C) and microwave–vacuum (MV) drying methods; Table S2: Average pore area in pasta with varying levels of *Rosa rugosa* pomace, obtained using convective (C) and microwave–vacuum (MV) drying methods; Table S3: Correlations between the analyzed parameters. Red color indicates significant correlation (*p* < 0.05).
