# Supplementary material for: The Effect of the Incorporation Level of Rosa rugosa Fruit Pomace and Its Drying Method on the Physicochemical, Microstructural, and Sensory Properties of Wheat Pasta
Source: Molecules. 2025 Jul 29;30(15):3170. doi: 10.3390/molecules30153170 (PMC12348497; doi:10.3390/molecules30153170)
Supplement: Supplementary file 1 [file molecules-30-03170-s001.zip › molecules-3769741-supplementary.pdf]

Table S1. Cooking time of pasta with varying levels of *Rosa rugosa* pomace, obtained using convective (C) and microwave–vacuum (MV) drying methods.

Pomace content (%)	Drying method	Cooking time (min)
0		2.5±0.0
2		2.5±0.0
4	C	2.5±0.0
6		2.5±0.0
8		2.5±0.0
0		1.5±0.0
2		1.5±0.0
4	MV	1.5±0.0
6		1.5±0.0
8		1.5±0.0

Table S2. Average pore area in pasta with varying levels of *Rosa rugosa* pomace, obtained using convective (C) and microwave–vacuum (MV) drying methods.

Pomace content (%)	Drying method	Average pores aere (mm^2)
0	C	0.102±0.060^d
2		0.335±0.074^e
4		0.367±0.019^f
6		0.368±0.002^f
8		0.362±0.023^f
0	MV	0.059±0.038^c
2		0.032±0.013^a
4		0.042±0.038^b
6		0.039±0.017^b
8		0.104±0.092^d

Two-factor analysis of variance

Factor	*p-value*
**Method of drying**	<0.001*
**RFP of addition**	<0.001*
**Type of drying × RFP of addition**	<0.001*

$^{a–g}$ – homogeneous groups ($p < 0.05$); * statistically significant ($p < 0.05$).

Table S3. Correlations between the analyzed parameters. Red color indicates significant correlation ($p < 0.05$).

Parameters	Weight increase index	Cooking losses	Elasticity	Hardness	Gumminess	Surface area	Percent object volume	St.Th	Total por.	DA	Appearance	Smell	Taste	Texture	Overall
Weight increase index	1,000														
Cooking losses	-0,608	1,000													
Elasticity	0,173	-0,803	1,000												
Hardness	0,151	-0,810	0,894	1,000											
Gumminess	0,174	-0,831	0,960	0,961	1,000										
Surface area	-0,394	-0,081	0,383	0,441	0,383	1,000									
Percent object volume	-0,801	0,787	-0,386	-0,381	-0,392	0,407	1,000								
St.Th	-0,711	0,709	-0,256	-0,344	-0,304	0,355	0,939	1,000							
Total por.	0,801	-0,787	0,386	0,381	0,392	-0,407	-1,000	-0,939	1,000						
DA	-0,594	0,649	-0,260	-0,315	-0,264	0,261	0,879	0,965	-0,879	1,000					
Appearance	-0,048	0,661	-0,767	-0,714	-0,760	-0,458	0,335	0,388	-0,335	0,427	1,000				
Smell	0,189	-0,622	0,759	0,824	0,792	0,319	-0,209	-0,067	0,209	-0,025	-0,262	1,000			
Taste	0,210	-0,812	0,938	0,954	0,978	0,460	-0,328	-0,265	0,328	-0,228	-0,753	0,813	1,000		
Texture	0,197	-0,787	0,914	0,922	0,925	0,576	-0,327	-0,296	0,327	-0,292	-0,855	0,686	0,958	1,000	
Overall	0,300	-0,709	0,788	0,840	0,852	0,494	-0,319	-0,321	0,319	-0,272	-0,773	0,620	0,906	0,926	1,000

St.Th- **Structure thickness (mm); Total por. - Total porosity (%); DA- Degree of anisotropy (-)**
